# Applications of Saponin Extract from Asparagus Roots as Functional Ingredient

**DOI:** 10.3390/foods13020274

**Published:** 2024-01-15

**Authors:** Amel Hamdi, Isabel Viera-Alcaide, Ana Jiménez-Araujo, Rocío Rodríguez-Arcos, Rafael Guillén-Bejarano

**Affiliations:** 1 Instituto de la Grasa, Consejo Superior de Investigaciones Científicas (CSIC), Pablo de Olavide Universitary Campus, Building 46, Carretera de Utrera Km 1, 41013 Seville, Spain; amelhamdi1988@yahoo.fr (A.H.); iviera@ig.csic.es (I.V.-A.); rrodri@ig.csic.es (R.R.-A.); rguillen@ig.csic.es (R.G.-B.); 2 Molecular Biology and Biochemical Engineering Department, Centro Andaluz de Biología del Desarrollo (CABD), University Pablo de Olavide (UPO), CSIC/UPO/JA, Carretera de Utrera Km 1, 41013 Sevilla, Spain

**Keywords:** bio-emulgent, foaming properties, saponins, steroid, *Quillaja*, asparagus root, agricultural byproduct, sustainability, *Tribulus*, pancreatic lipase inhibition

## Abstract

When replanting an asparagus field, the roots of the previous crop are crushed and incorporated into the soil, creating problems of autotoxicity and fungal infections. Asparagus roots can be considered as a valuable byproduct, since they are very rich in saponins (3–6%), compounds currently considered as bio-emulsifiers. The objective is to evaluate the emulsifying and foaming capacity of a saponin extract from asparagus roots (ARS) and compare it with other commercial extracts. ARS was obtained using a process patented by our research group. The results have shown that ARS has activity similar to *Quillaja* extract. Its critical micellar concentration falls between that of *Quillaja* and *Tribulus* extracts (0.064, 0.043, and 0.094 g/100 mL, respectively). Both emulsifying and foaming activities are affected by pH, salt, and sucrose to a similar extent as the other extracts. Additionally, it has demonstrated an inhibitory effect on pancreatic lipase, which is even better than the other two studied extracts, as indicated by its IC_50_ value (0.7887, 1.6366, and 2.0107 mg/mL for asparagus, *Quillaja*, and *Tribulus*, respectively). These results suggest that ARS could serve as a natural emulsifying/foaming agent for healthier and safer food products and as a potential aid in treatments for obesity and hyperlipidemia.

## 1. Introduction

The food and cosmetics industries, perhaps driven by evolving consumer trends, are increasingly seeking natural additives to replace synthetic ones. One of the areas generating significant interest is emulsions. Emulsifiers, like all surfactants, have a chemical structure with a hydrophilic and a lipophilic part. This characteristic reduces the surface tension of the liquids forming the emulsion, promotes the dispersion of droplets, and creates a protective layer around them that enhances long-term stability and inhibits aggregation [1]. These properties also make them effective foaming agents. In the food industry, and even more so in the cosmetics and detergent manufacturing industries, synthetic emulsifiers, which are known for their toxicity [2], are still used. Consumers are becoming increasingly aware of the risks that these products present, not only to their health but also to the environment. This is due to the pollution that occurs during their production and their release into the environment after use [3]. The environmental impact of these compounds is more extensive than one might think, as surfactants, including emulsifiers and foaming agents, contaminate groundwater [4], affect microorganisms [5], flora [6], and fauna (shellfish, fish, insects, and mammals) [3], not to mention their cumulative effects due to their low biodegradability [7]. For all these reasons, the search for natural compounds (biosurfactants and bio-emulsifiers) that can replace these synthetic additives is of great current interest.

Saponins, as the etymology suggests (the word “saponin” comes from the Latin “saponinus” and means “related to soap”), have the ability to produce a soapy layer and foam when agitated in an aqueous medium. They are naturally present in a wide range of plant-based foods (soybean, oat, alfalfa, *Allium* species, asparagus, spinach, tea, potato, etc.) [8]. Their chemical structure consists of a central core (sapogenin, genin, or aglycone) of a steroid or triterpenic nature and a series of sugar chains (one, two, or three) that attach to different points on it, giving rise to mono-, bi-, or tridesmosidic saponins [8]. The aglycone has a lipophilic character, while the sugar chains are hydrophilic. This amphiphilic character makes saponins effective emulsifying and foaming agents. In fact, they are already considered as bioemulsifiers [3], and extracts from the *Quillaja saponaria* Molina plant are recognized as natural humectants, emulsifiers, and foaming agents (E999i, E999ii) [2]. The search for new sources of saponins is of great interest because their technological properties are related to their chemical structure. The emergence of new saponins could expand the range of applications for these natural compounds.

In addition to their technological functionality, saponins also have biological activity as hypocholesterolemic, antifungal, anti-obesity, anti-diabetic, antihypertensive, and anti-inflammatory agents [8,9,10]. They also demonstrate immune-stimulating and anticancer activity [11,12]. This bioactivity is also dependent on their chemical structure, so changes in the aglycone or in the number and/or length of their sugar chains could result in changes in their bioavailability and bioactivity.

It is widely known that saponins are present in a wide variety of plant-based foods, including asparagus [8]. In this vegetable, saponins are found in all parts of the plant, not just in its edible portion, with the fruits and roots being particularly rich sources. While in the asparagus spear of commercial varieties (*Asparagus officinalis* L.), protodioscin is the predominant saponin (with diosgenin as the aglycone and two sugar chains containing four sugar residues), specific genotypes of “triguero” asparagus from the Huétor-Tájar landrace [13,14], other species of asparagus (*A. albus*, *A. acutifolius*, *A. maritimus*, *A. pseudoscaber*) [15,16,17], and the roots [16,17] have been described as having a wide structural variety of saponins.

During asparagus cultivation, a large number of roots are produced as a byproduct. This occurs because when the productivity of the plantation decreases, it is necessary to uproot it and replant new ones. This practice happens every 10–12 years, with the remains of the roots and rhizomes being ground up and incorporated into the soil. The reasons for the phenomenon known as “asparagus decline” could be found in this practice. This phenomenon involves a decrease in crop yield over time and the inability to replant a particular piece of land for at least 5 years after the initial planting [18]. This limits the commercial lifetime of the plantation and leads to a continuous search for new lands for cultivation. The ultimate causes of this process may include the presence of certain fungi, primarily *Fusarium* [19], and certain allelopathic substances exuded by the root [20]. Some authors even argue for the combined effect of these two factors [21]. Therefore, removing the roots from agricultural land would be a significant step in combating this phenomenon. It would be of great interest to the agricultural sector to find added value for this byproduct, making it profitable for the industry. The average saponin content in asparagus roots is between 3 and 6% of their dry weight [16,17,22,23], while the content of other plant products known as saponin sources (such as yucca, ginseng, and oat bran) ranges between 4 and 9% [24]. Our research group has published a patent for extracting and purifying bioactive compounds from asparagus [25], which is easily applicable to industrial processes and environmentally friendly. Obtaining a saponin extract from asparagus roots through this method would be a significant advancement in adding value to this byproduct and, consequently, in the sustainability of asparagus cultivation.

Therefore, the objective of this study is to evaluate the emulsifying and foaming abilities of a saponin extract from asparagus roots in comparison to other commercial saponin-rich extracts. After chemical characterization, the extracts will be tested under different conditions of concentration, pH, and the presence of additives to discuss their potential use in formulating “green-label” foods, i.e., without synthetic additives. Another highly interesting activity that will be studied is the inhibition of pancreatic lipase, which is related to the hypocholesterolemic and anti-obesity effects associated with saponins. The results of this study will be of great technological interest as well as from a health perspective. By incorporating asparagus saponins into food formulations, safer and healthier foods can be achieved while also helping the asparagus industry to have more sustainable and environmentally friendly production.

## 2. Materials and Methods

### 2.1. Obtaining Saponin Extracts

Asparagus roots were collected from production fields in Huétor-Tajar, Granada, Spain, during the fall of 2019. The samples were sent to the Instituto de la Grasa—CSIC, where they were cleaned to remove excess dirt and other impurities. Once cleaned, they were extracted according to the patent WO2011/161293 A1 [25]. In brief, the roots (10 kg) were treated in an autoclave (Steriflow, Madinox, Barcelona, Spain) at 121 °C for two hours with a root-to-water ratio of 1:2. Afterward, the mixture was filtered, and the aqueous extract was loaded into a column filled with Amberlite XAD adsorption resin (Rohm and Hass, Spain, S.A.). Following sample injection, the resin was washed with a column volume of water, and saponins were subsequently eluted by washing with two column volumes of 96% ethanol. This ethanolic fraction was concentrated and lyophilized. The resulting dry residue was considered asparagus root saponin extract (ARS). The primary benefit of this process is that the operating conditions are commonly used in the industrial canning of plant foods, with some necessary adjustments. Additionally, the only organic solvent employed is ethanol, which is authorized for use in the food industry. Given its cost and low toxicity, ethanol is extensively utilized.

The *Quillaja* saponin extract (QS) was obtained from Sigma (S4521) and is stated to have a sapogenin content of >10%. The *Tribulus terrestris* saponin extract (TS) was purchased from a health food store. This packaging includes nutritional labeling and a stated saponin content of 90%. QS was selected from Sigma because it is a purified extract and could serve as a good comparison with ARS, given that quillaja saponins are widely used in cosmetics, food, and medicine. TS was chosen for this comparative study because it is an unpurified extract and is in high demand in the dietary sector. In summary, both extracts have been chosen to represent two extremes in the commercial applications of saponins, between which the saponin extract from asparagus roots could be positioned.

### 2.2. Chemical Characterization of the Extracts

In order to obtain equivalent information for all the extracts, chemical characterization was performed, which involved determining the content of saponins, phenolic compounds, proteins, and ash.

#### 2.2.1. Saponin Content

The saponin content evaluation was conducted following the methodology described by Vázquez-Castilla et al. [14]. An HPLC system from Waters Alliance in Manchester, UK, was used. This system was equipped with a Mediterranean Sea C18 reverse-phase analytical column with the following specifications: 25 cm in length, 4.6 mm inner diameter, and 5 μm particle size. The analysis involved the use of an elution gradient with two solvents, solvent A (water with 1% formic acid) and solvent B (acetonitrile with 1% formic acid). The elution gradient program was as follows: 0–30 min with 20% B, 30–60 min with a linear gradient to 30% B, 60–70 min with a linear gradient to 100% B, and 70–80 min with a linear gradient back to 20% B. Detection of saponins was carried out using an online connected quadrupole mass analyzer (ZMD4, Micromass, Waters, Inc., Manchester, UK). The flow in the mass spectrometer was controlled using a split with a flow rate of 0.3 mL/min. For mass spectrometry analysis, ESI (Electrospray Ionization) mass spectra were recorded at ionization energies of 50 and 100 eV in the negative mode and 50 eV in the positive mode, covering the *m*/*z* range from 200 to 1200. The capillary voltage was set at 3 kV, the desolvation temperature was 200 °C, the source temperature was 100 °C, and the extractor voltage was 12 V. For saponin content quantification, two different external standards, protodioscin and shatavarin IV, were used. For each standard, ten dilutions ranging from 0 to 500 μg/mL were prepared and injected into the LC-MS system. The selected ion chromatogram corresponding to the molecular ion of each standard in negative mode at 50 eV was integrated, and the peak area was plotted against the concentration and subjected to regression analysis, allowing the determination of saponin content in the samples.

#### 2.2.2. Phenolic Compounds by Folin–Ciocalteu Assay

The total phenolic content was assessed through the Folin–Ciocalteu spectrophotometric method and was quantified and expressed as a percentage of gallic acid equivalent [26].

#### 2.2.3. Phenolic Compounds by HPLC-DAD

Solutions of each sample at a concentration of 10 mg/mL were subjected to high-performance liquid chromatography (HPLC) analysis [16]. The HPLC system used was a Jasco-LC-Net II ADC liquid chromatograph system equipped with a diode array detector (DAD). Separation of phenolic compounds was achieved using a Mediterranea Sea C18 reverse-phase analytical column with the following specifications: 25 cm in length, 4.6 mm inner diameter, and 5 μm particle size. The gradient profile was created using two solvents, solvent A (water with 1% formic acid) and solvent B (acetonitrile with 1% formic acid). The gradient program was as follows: from 0% B to 20% B over the first 20 min, followed by an increase to 21% B over the next 8 min, holding at 21% B for 2 min, then increasing to 30% B over the next 10 min, followed by a raise to 100% B over the next 5 min, and finally holding at 100% B for 5 min. The flow rate was set at 1 mL/min, and the column temperature was maintained at 30 °C. Spectra for all peaks were recorded in the 200–600 nm range, and the chromatograms were acquired at 360 nm. The quantification of individual phenols (caffeic, p-coumaric, t-ferulic, and coumaryl glycerol acids) was accomplished using an eight-point regression curve within the range of 0–250 μg, based on standard compounds.

#### 2.2.4. Protein Concentration

The concentration of protein was determined by calculating the % nitrogen content multiplied by 6.25, using elemental microanalysis. This analysis was performed using a Leco CHNS932 analyzer (St. Joseph, MI, USA) [27] with a burning temperature of 950 °C and an afterburner temperature of 850 °C.

#### 2.2.5. Ash Content

The ash content was determined by incinerating samples in a muffle furnace (Nabertherm model B180, Bremen, Germany) at 550 °C [27] until white ash was obtained.

### 2.3. Critical Micelle Concentration Assay (CMC)

For this determination, the method of solubilization of the liposoluble dye Sudan III was employed, following the procedure described by Schreiner et al. [28]. In summary, dilutions of each sample ranging from 0.005 to 0.5 g/100 mL were prepared. From each dilution, 0.5 mL was taken and 20 μL of the Sudan III solution in hexane was added. The mixture was vigorously stirred for 1 min and then left to stand for 30 min. Absorbance was read at 490 nm using an iMark plate reader (Bio-Rad, Hercules, CA, USA). Each sample was tested at least in duplicate. The obtained absorbance values are plotted against concentration. At concentrations below the CMC, absorbance readings are low because the dye is not soluble in the aqueous medium. However, once the CMC is exceeded, significant increases in absorbance are observed as Sudan III incorporates into the micelles formed by emulsion. The CMC is calculated as the point of intersection of the regression lines for the two regions (above and below the CMC).

### 2.4. Determination of Emulsifying Capacity and Emulsion Stability

To determine the emulsifying capacity, the Wang and Kinsella method [29] was used with some modifications. All three samples (ARS, QS, and TS) were tested at different concentrations ranging from 0.01 to 0.5 g/100 mL. In a glass test tube, 5 mL of each solution was mixed with 3.3 mL of commercial sunflower oil and stirred using a VWR homogenizer, model VDI 12 (Radnor, PA, USA), at a speed of 20,000 rpm for one minute. Subsequently, the tubes were centrifuged at 1300× *g* for 5 min. The emulsifying capacity (%) was expressed using the following formula:

Emulsifying capacity = (H_e_/H_t_) × 100 (1)

where H_e_ is the height of the emulsified layer (cm), and H_t_ (cm) is the height of the total tube content. Each sample was tested at least in duplicate.

To determine the stability of the formed emulsion, the same procedure as described above was followed, but before centrifugation, the emulsion was subjected to thermal treatment by immersing the tubes in a thermostatic bath at 80 °C for 30 min. The stability (%) is calculated using the following formula:

Emulsion stability = (H_h_/H_t_) × 100 (2)

where H_h_ is the height of the emulsified layer after heating (cm), and H_t_ is the height of the total tube content (cm). Each sample was tested at least in duplicate.

### 2.5. Determination of Foaming Capacity and Foam Stability

The Cano-Medina et al. method [30] was employed with some modifications. In this case, the concentration range used was between 0.03 and 0.5 g/100 mL. Three milliliters of each concentration were dispensed into glass tubes and stirred using a VWR homogenizer, model VDI 12, at a speed of 40,000 rpm for one minute. The initial foam layer height was measured, as well as the height after 2, 4, and 24 h. The data presented here corresponds to a 24 h time point (H_24_), as this is when the most significant differences were observed. The foam capacity and foam stability were calculated using the following formulas:

Foaming capacity = (H_f_ − H_0_/H_0_) × 100 (3)

Foam stability = (H_24_ − H_0_) × 100/(H_f_ − H_0_) (4)

where H_0_ is the initial height (cm), H_f_ is the height of the total tube content after stirring (cm), and H_24_ is the height of the total tube content after 24 h at room temperature (cm). Each sample was tested at least in duplicate.

### 2.6. Influence of Environmental Conditions (pH, Presence of Sodium Chloride, and Sucrose) on Emulsifying and Foaming Properties

The effect of pH on the emulsifying and foaming properties of saponin extracts has been tested at two concentration levels, 0.03 and 0.5 g/100 mL. Various pH values ranging from 2 to 10 were tested, and the pH of the solutions was adjusted using 0.01 M HCl or NaOH as necessary. The natural pH of the saponin solution was approximately 7.8. Additionally, the presence of 1 M sodium chloride and 5% sucrose, which are common additives in the food industry, was tested.

The emulsifying and foaming property tests were conducted as previously described in Section 2.4 and Section 2.5.

### 2.7. Determination of the Inhibitory Effect of Saponin Extracts on Pancreatic Lipase

Pancreatic lipase plays a crucial role in lipid metabolism, making it a widely employed model to calculate the effectiveness of natural products in combatting obesity [31]. The evaluation of pancreatic lipase inhibitory activity by the various extracts under investigation, as well as the positive control Orlistat, was carried out using p-nitrophenyl butyrate (p-NPB) as the substrate, following the procedure described by Jaradat et al. [32].

Saponin samples were dissolved in Tris-HCl buffer, which consisted of 100 mM Tris-HCl and 5 mM CaCl_2_, adjusted to a pH of 7. Then, 160 µL of increasing concentrations of the three saponin extracts and 20 µL of pancreatic lipase (20 mg/mL in Tris-HCl buffer) were combined within the microplate wells. The microplate was subsequently incubated at 37 °C for 30 min, followed by the addition of 20 μL of p-NPB substrate (10 mM in dimethylformamide). The activity of pancreatic lipase was determined by measuring the hydrolysis of p-NPB to p-nitrophenol at a wavelength of 405 nm through kinetic readings employing a microplate reader (i-Mark from Bio-Rad). This process was replicated for the different extracts and for Orlistat.

The percentage of inhibition was computed using the following formula:

% inhibition = (A − B) × 100/A(5)

where A represents the lipase activity in the reaction solution without the saponin extract, and B represents the lipase activity in the presence of the extract.

All measurements were conducted in triplicate, and the IC_50_ values, denoting the concentration that inhibits pancreatic lipase activity by 50%, were determined from concentration-dependent inhibition curves.

### 2.8. Statistical Analysis

In the study, all samples were analyzed at least in duplicate to ensure the reliability of the results. An analysis of variance (ANOVA) was performed to assess the differences among samples. This statistical analysis helps determine whether there are significant differences between the groups being compared. It was conducted using the Statgraphics^®^ Centurion Version XVI.I. To distinguish between the means, the Fisher’s Least Significant Difference (LSD) procedure was employed. The LSD test is used to identify which means significantly differ from each other. The significance level used for all statistical tests was set at *p* < 0.05, which means that differences were considered statistically significant if the probability of their occurrence by chance was less than 5%.

For regression analysis, the method of simple regression was chosen. Various curvilinear models were fitted to the data to understand the relationships and trends within the dataset. The same software program (Statgraphics^®^ Centurion Version XVI.I) was used for this analysis, and the significance level remained at *p* < 0.05.

These statistical methods were employed to draw conclusions, make comparisons, and identify any relationships or trends within the dataset, ensuring that the findings were statistically supported.

## 3. Results

### 3.1. Characterization of the Different Saponin Extracts

The saponin content is not the exclusive component that can interact with the properties being studied in this work. Protein, phenol, and ash content can have a significant influence, so their determination is of great interest. ARS was obtained by applying Guillén-Bejarano et al.’s patent [25], yielding a 4.18% average on a dry-weight basis, and it has been fully characterized in our laboratories as described in the Materials and Methods chapter. In Sigma’s catalog, the only information provided regarding their product S4521—*Quillaja* bark saponin (QS)—is that it contains over 10% of saponins, expressed as sapogenin. TS, acquired from a health food store, comes with comprehensive nutritional information on its labeling. The saponin, phenol, protein, and ash contents of the three samples are presented in Table 1.

**Figure 1:**
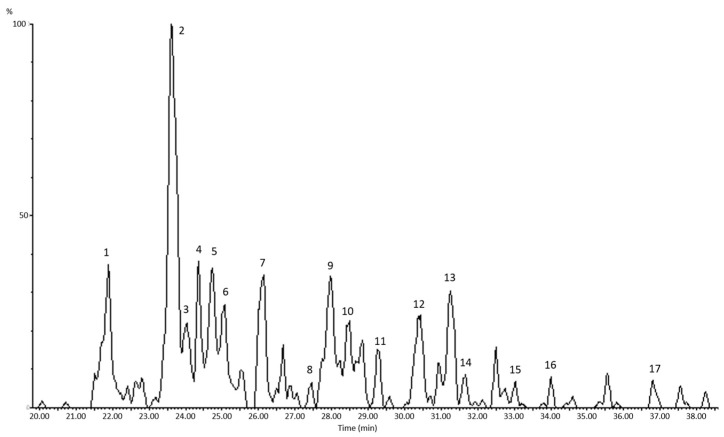
The total ion chromatogram (TIC) of HPLC-MS analysis of asparagus root saponin extract (ARS). The peaks identified as saponins by their MS spectra are numbered consecutively from 1 to 17.

The saponin content varied among the different samples. In ARS, up to 17 different saponins were identified by HPLC-MS (Figure 1), most of which have been previously described in various varieties and species of the *Asparagus* genus [13,14,16,17]. The aglycone present is sarsasapogenin, which has a steroid structure with 1–4 sugar residues attached at one or two positions on the steroid ring (mono- and bidesmosidic saponins). This content of 47.64% is expressed based on saponin, considering both the sapogenin content and the sugar residues attached to it. An approximate content expressed in genin for ARS would be 20.22%, considering sarsasapogenin as the aglycone and an average content of three sugar residues in the molecule. This percentage is comparable to the one presented in Table 1 for QS, so it can be said that ARS has roughly twice the saponin content of QS.

According to the literature, *Quillaja* saponins are of a triterpenic nature. Their aglycone is quillaic acid, and they have a wide variety of sugar chains. Up to 100 different structures have been described, with up to seven sugar units, one glucuronic acid unit, and one fatty acid unit [11,33]. In summary, *Quillaja* saponins are triterpenic and mostly bidesmosidic. The manufacturer of TS does not specify whether the content of 12% is expressed in terms of aglycone or saponin, so it cannot be compared with ARS and QS, at least at this point in the discussion. The composition of the *T. terrestris* extract is also very complex, with more than 30 different saponin structures described. Among them, the most abundant are protodioscin and protogracillin, which are steroid saponins, both mono- and bidesmosidic, containing diosgenin as the aglycone [34,35].

The phenol content, quantified using the Folin–Ciocalteu method (Table 1), was much lower than the saponin content in the case of ARS and QS but fell within the same range as saponins in TS. When ARS was studied using HPLC-DAD, only 0.65% of the phenolics was quantified, with caffeic acid being the major component (over 50%), and coumaryl glycerol, p-coumaric acid, and t-ferulic acid also present. These compounds had been previously described in *A. acutifolius* [17]. According to Sigma’s labeling for QS, the extract has been purified to remove low molecular weight contaminants, which explains the low phenol content and other compounds in the extract. TS is the richest in phenols, which might indicate a lower degree of purification. Flavonoids such as kaempferol, astragalin, kaempferol-3-rhamnoglycoside, tribuloside, and rutin have been identified in the literature, quantifying at approximately 1.5 times the saponin content [34,35]. This information might suggest that the TS sample could have a lower degree of purification than ARS and QS.

ARS has a high protein content, exceeding 10%. Proteins are widely known as emulsifying and foaming agents [36], so their presence in this extract could have synergistic effects with saponins. The ash content of the three samples tested is less than 1%, so its impact on subsequent tests will be very limited.

### 3.2. Emulsifying Properties

#### 3.2.1. Critical Micelle Concentration Assays

**Figure 2:**
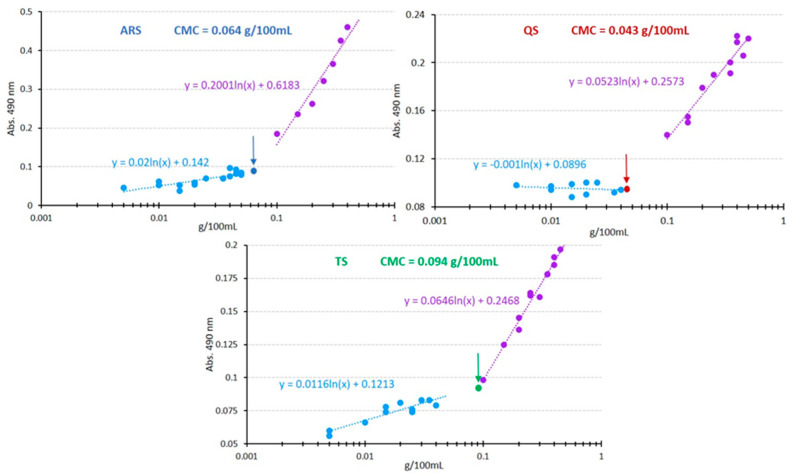
Graphical representation of Sudan III absorbance as a function of saponin extract concentration, displayed on a logarithmic scale. The CMC is distinctly indicated by an arrow for each sample: navy blue for asparagus root saponin extract (ARS), red for quillaja saponin extract (QS), and green for Tribulus saponin extract (TS). Light blue points indicate concentrations below critical micelle concentration (CMC), and purple ones those beyond CMC.

The CMC values were determined through the Sudan III assay, and the results are depicted in Figure 2. For each saponin extract, two distinct zones were observed: at low concentrations (represented by the light blue points), small increases in absorbance were observed with increasing concentration. However, beyond a certain point (the CMC), a sudden and notable increase in absorbance occurred (indicated by the purple points). The CMC was calculated as the point of intersection between both regression lines and was visually marked in Figure 2 with an arrow.

QS exhibited the lowest CMC value, measuring at 0.04 g/100 mL. This value falls within the range reported by other authors in the literature, from 0.005 to 0.77 g/L [28,37]. Conversely, TS displayed the highest concentration at 0.094 g/100 mL. Although *Tribulus* has been less extensively studied as a saponin source compared to *Quillaja*, the values we found in the literature for its CMC (ranging from 0.048 to 0.106 g/L) [1,28] align with the findings presented in this study. ARS, the asparagus root saponin extract, exhibited a CMC of 0.064 g/100 mL, positioning it between QS and TS. It is widely known that the lower the CMC, the higher the emulsifying capacity. It is important to note that CMC and the emulsifying and foaming capacities are not solely dependent on saponin concentration. While the saponin concentration in ARS is double that in QS, it seems that their different chemical structure has a significant influence on the CMC, with QS having a lower CMC than ARS. In this regard, the presence of proteins in ARS could be positively influencing the determined CMC value in this assay.

#### 3.2.2. Influence of Concentration on Emulsifying Properties

**Figure 3:**
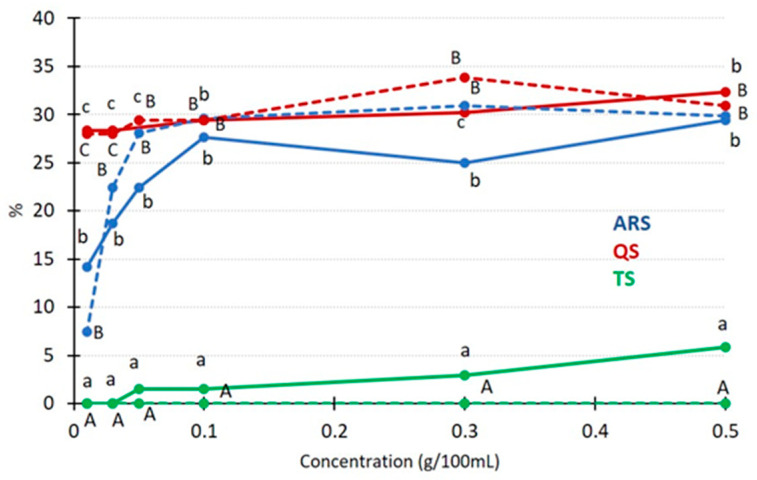
The effect of the saponin concentration on emulsifying capacity and stability. Solid lines represent capacity, while dotted lines represent stability. If the same letter is used to denote different data points, it indicates that there are no significant differences at the same concentration (lowercase for capacity and uppercase for stability), with *p* < 0.05.

Various concentrations were evaluated, including 0.03, 0.05, 0.1, 0.3, and 0.5 g/100 mL, for each of the saponin types under investigation (ARS, QS, and TS). Figure 3 illustrates the impact of concentration on both the emulsifying capacity and the stability of the resulting emulsions.

ARS exhibited a significant increase in both capacity and stability as the concentration increased. At concentrations below 0.1 g/100 mL, ARS demonstrated lower values for both studied parameters compared to QS. However, beyond this concentration, ARS and QS yielded very similar results. These differences in performance may be attributed to the distinct chemical structures of asparagus saponins (steroid) and *Quillaja* saponins (triterpenoid). Triterpenoid aglycones, as found in *Quillaja* saponins, are known to be the best in forming and maintaining emulsions due to their ability to create highly viscoelastic networks [33]. When working with TS (steroid saponin), notably lower results were obtained, probably related to its low saponin concentration. TS displayed a capacity of around 5% at the highest concentration, in patent contrast to the approximately 30% capacity observed for ARS and QS. Furthermore, emulsions formed with TS proved to be unstable after heating and centrifugation. These findings align with the results obtained by Schreiner et al. [28], who compared hydroethanolic extracts of *T. terrestris* and commercial *Quillaja* bark saponin, reporting similar trends. These results also support the idea of a low degree of purification of the TS sample, as mentioned in the discussion of Table 1. *Tribulus* saponin exhibited lower capacity and stability compared to *Quillaja* bark saponin, although the differences in the origin and composition of the two *Tribulus* samples might account for the variation observed. In another study, triterpenoid saponins extracted from *Camellia* seed byproduct were evaluated parallel to *Quillaja* saponins and Tween 80^®^, a synthetic emulsifier [38]. While Tween 80^®^ exhibited the best performance, *Camellia* saponins demonstrated behavior very similar to *Quillaja*, a well-recognized natural emulsifier. This highlights the potential of obtaining saponins from agricultural byproducts as a sustainable and viable alternative.

#### 3.2.3. Influence of pH

**Figure 4:**
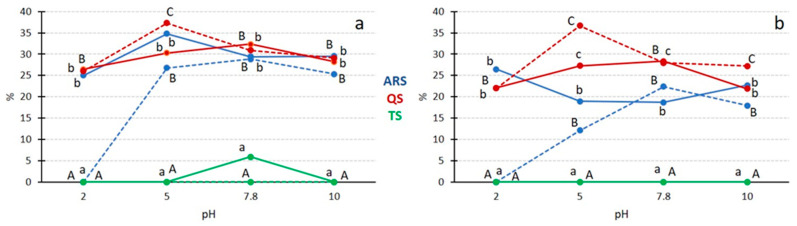
The influence of pH value on saponin emulsifying capacity and emulsion stability. Solid lines represent capacity, while dotted lines represent stability. If the same letter is used to denote different data points, it indicates that there are no significant differences at the same pH value (lowercase for capacity and uppercase for stability), with a *p* < 0.05. (**a**) The saponin extract concentration is 0.5 g/100 mL. (**b**) The saponin extract concentration is 0.03 g/100 mL.

The pH value plays a crucial role in influencing both emulsifying parameters as it can lead to changes in electrostatic repulsion, which prevents the aggregation and coalescence of emulsion droplets [1]. Figure 4 visually illustrates the impact of pH values on emulsifying capacity and stability.

At the 0.5 g/100 mL concentration level (Figure 4a), ARS and QS exhibited similar results. Over the entire range of pH values, the emulsifying capacity of both extracts fell within the 25–35% range, with no significant differences between them, except at pH 2, where both extracts showed slightly lower results. However, the stability of emulsions was affected by pH values. Within the pH range of 5–10, high stability levels were observed, but at pH 2, a decrease in stability occurred, with QS forming more stable emulsions than ARS. This behavior can be attributed to the chemical structure of the main component of *Quillaja* saponin, which contains a triterpenoid aglycone with glucuronic acid in its structure [39]. At pH values lower than the pKa (3.5), the carboxylic group becomes fully uncharged, resulting in reduced electrostatic repulsion between droplets, leading to their aggregation [1,38,40]. Although no ionic saponins have been described in asparagus to date, the numerous -OH groups in their sugar moiety and the higher concentration of saponin in this extract could lead to similar behavior for ARS. The optimal results in terms of capacity and stability were obtained for both ARS and QS at pH 5–7.8, but both parameters decreased in basic media (pH 10), possibly due to the hydrolysis of glycosidic and ester linkages, leading to lower emulsifying capacity and stability [41]. TS exhibited very low capacity, only at pH 7.8 (5.3%). While TS, like ARS, is also steroidal, the low concentration of saponins in commercial extracts may account for its low capacity.

At the 0.03 g/100 mL concentration (Figure 4b), differences between QS and ARS became apparent. QS displayed the expected behavior, with the highest capacity near neutral pH and the highest stability at pH 5. ARS had a lower emulsifying capacity than QS, except for the value obtained at pH 2, which was significantly higher for ARS. The presence of proteins in ARS may have influenced its results at low concentrations. The stability of ARS emulsions followed the general pattern, with the most stable emulsion at pH 7.8 but decreasing stability as the pH values moved toward acidity or alkalinity. TS did not exhibit any significant activity.

*Quillaja* saponins, known under the commercial trademark Q-naturale^®^ from Ingredion (Westchester, IL, USA), are widely used as natural food emulsifiers. In this study, ARS showed similar behavior to QS but at higher concentrations. This suggests that asparagus root extract could be considered a promising natural emulsifier. Its stability within the pH range typically found in foods makes it a suitable candidate for inclusion in food formulations, potentially reducing the need for synthetic emulsifiers like Tween^®^.

#### 3.2.4. Influence of Other Additives

In the formulation of foods, it is common to include various additives, and among them, sodium chloride (NaCl) and sucrose are frequently used. Their presence in solutions can alter the performance of emulsifiers because they affect the ionic environment of the food system. In our study, we examined the impact of 1 M NaCl and 5% sucrose, as detailed in Table 2.

When working with more concentrated solutions of saponin extracts (0.50 g/100 mL), ARS and QS did not exhibit significant differences (indicated by lowercase letters in Table 2) in emulsifying capacity, except in the presence of sucrose, where ARS displayed a higher capacity than QS. In contrast, TS consistently had the lowest capacity across all conditions. At lower concentrations (0.03 g/100 mL), QS emerged as the best emulsifier when paired with both sodium chloride and sucrose as additives. These trends were mirrored in emulsion stability as well.

When comparing the impact of the additives (indicated by capital letters in Table 2), it can be concluded that sodium chloride increased the capacity of each extract but decreased stability. A study by Yang et al. [42] on commercial *Quillaja* extract (Q-Naturale^®^) observed a similar trend, with no coalescence observed up to 400 mM of NaCl, but above this concentration, droplet flocculation occurred, likely due to increased attractive forces between droplets (e.g., van der Waals forces) and decreased repulsive forces (e.g., electrostatic repulsion) [1,42]. Yucca (*Yucca schidigera*) saponins showed similar behavior under high ionic strength and low pH conditions [1]. In this context, the decrease in stability observed in the current study was expected, given the much higher NaCl concentration tested (1 M).

The presence of sucrose in the medium also resulted in significant differences in capacity and stability, albeit to a lesser extent than NaCl. In general, sucrose either decreased the capacity or had no significant effect on this property, and stability remained mostly unchanged. Since the sugar moiety is partially responsible for the emulsifying properties of saponins, the increase in sugar concentration did not substantially affect this property of saponins, consistent with findings in the literature. For example, emulsions with *Saponaria officinalis* root extracts increased their stability with the addition of 1% sugar [43], while 2% and 4% sugar did not impact the stability of coffee creamer containing 2% *Quillaja* saponin [44]. In the latter study, the addition of sugar (1 or 2 tablespoons to 50 mL of hot black coffee) had no discernible effect on the appearance, particle size, microstructure, or color of the coffee/creamer emulsions.

It appears that the presence of salt or sugar in the medium affects ARS in a manner similar to how other natural saponins are affected. Consequently, saponins from asparagus could be considered a natural functional ingredient suitable for incorporation into various food systems.

### 3.3. Foaming Properties

#### 3.3.1. Influence of Concentration on Foaming Properties

**Figure 5:**
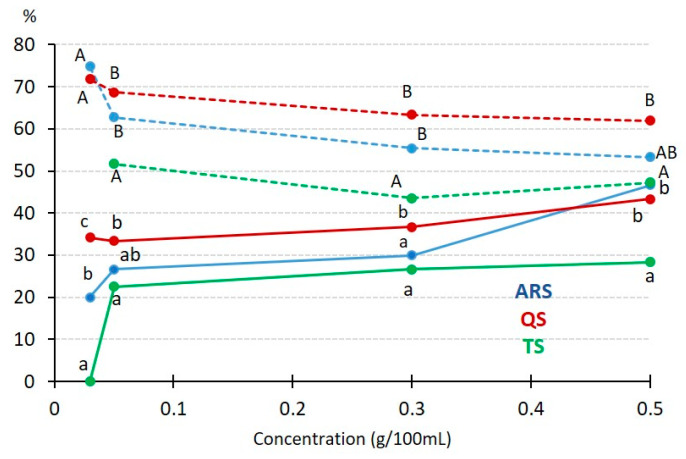
The influence of the saponin concentration on the foaming capacity and stability, solid lines representing capacity and dotted lines representing stability. If the same letter is used to indicate different data points, it signifies that there are no significant differences for the same concentration (lowercase for capacity and uppercase for stability) at a significance level of *p* < 0.05.

The foaming capability is an additional characteristic of saponins, where their chemical structure and surface activity in solution can have a positive impact on the formation and stability of dispersed systems [11]. In Figure 5, the foaming capacity and foam stability of the three saponin extracts tested at various concentrations are displayed.

**Figure 6:**
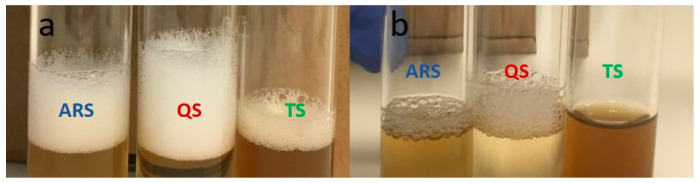
The appearance of the foam formed after agitating a 1 g/100 mL solution of the different saponins. (**a**) A total of 30 s after agitation. (**b**) A total of 24 h after agitation.

The concentration of saponin extracts had a distinct impact on both the foaming capacity and foam stability. The foaming capacity significantly increased with higher concentrations, while stability decreased as the concentration increased. Within the concentration range of 0.01–0.3 g/100 mL, QS exhibited the highest foaming capacity, but at 0.5 g/100 mL, there was no significant difference between QS and ARS. In terms of stability, QS and ARS displayed significantly better results compared to TS, with their foams being more stable than those of TS. The measurement of foam stability in this study was based on assessing the foam layer height, as described in the Materials and Methods section. Additionally, an increase in the bubble size was observed (Figure 6), indicating instability, although bubble size measurements were not included in this study. This effect aligns with observations made in other studies involving natural saponins [45].

Foaming capability indeed correlates with the chemical structure of saponins. Saponins with acidic groups or those with two sugar chains (bidesmosidic saponins) generally exhibit higher foaming ability compared to neutral or monodesmosidic saponins [11]. However, the behavior of *Tribulus* saponins (which consist of both steroid mono- and bidesmosidic saponins) can vary in the literature. Böttcher and Drusch [45] excluded this extract from their assays due to their low capability. On the contrary, Schreiner et al. [28] considered *Tribulus* extract as the most promising one among other natural emulsifiers. So, it is important to consider not only the chemical structure but also the saponin concentration and the presence of other compounds in the extract. In our assays, consistent with the findings in emulsion tests, TS displayed significantly lower foaming ability and stability, although not far from ARS. QS typically performed well in these tests [45,46], which aligned with the results obtained from ARS.

#### 3.3.2. Influence of pH

**Figure 7:**
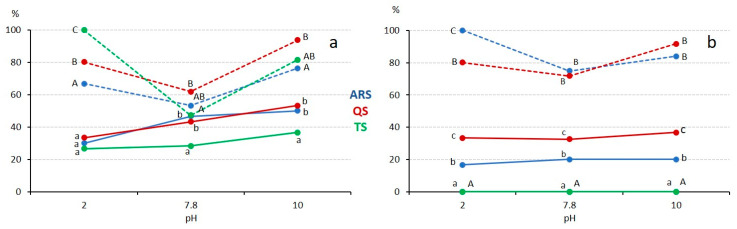
The impact of pH values on saponin foamability and foam stability, solid lines representing foam capacity and dotted lines representing foam stability. If the same letter is used to indicate different data points, it signifies that there are no significant differences for the same concentration (lowercase for capacity and uppercase for stability) at a significance level of *p* < 0.05. (**a**) The saponin extract concentration is 0.5 g/100 mL. (**b**) The saponin extract concentration is 0.03 g/100 mL.

The influence of pH values on foam formation and stability is evident in our results (Figure 7). At the concentration of 0.5 g/100 mL (Figure 7a), foaming capacity increased as pH levels rose, with ARS and QS achieving similar results at medium to high pH values. Interestingly, unlike emulsions, extreme pH values did not significantly affect foam stability; in fact, foam stability increased at these extreme pH values (2 and 10). This behavior contrasts with findings from other saponin extracts, such as *Xanthoceras sorbifolium* leaves [47] and *Glycyrrhiza glabra* roots [45], where no significant changes or increased stability were observed under extreme pH conditions. This suggests that the influence of the ionic state of carboxylic groups on foam stability may be less pronounced compared to its effect on emulsion stability.

At a low concentration of 0.03 g/100 mL (Figure 7b), TS did not exhibit any foam capacity at any pH value, and both ARS and QS showed no significant differences in foam capacity at different pH levels, with QS being more effective than ARS. The stability of the foam followed the same trends as observed at the higher concentration of 0.5 g/100 mL.

#### 3.3.3. Influence of Other Additives

The presence of NaCl and sucrose in the saponin solution had an impact on foam formation and stability, as presented in Table 3. At the concentration of 0.5 g/100 mL, ARS displayed the highest foamability with both additives and was not significantly affected by either of them. In contrast, QS showed a decrease in foam capacity when combined with NaCl and sucrose, while TS was affected by sucrose. Generally, at the lower concentration (0.03 g/100 mL), the foam capacity of all extracts decreased, with ARS and QS still exhibiting significantly higher foam capacity compared to TS. Solutions with NaCl yielded the best results at this concentration. The effect of increased ionic strength depends on the structural characteristics of saponin molecules. Ionic saponins, such as those from *Quillaja* bark, tend to be more affected than non-ionic ones like *Asparagus* and *Tribulus* saponins. The number of sugar chains, whether mono- or bidesmosidic saponins, also plays a role, with bidesmosidic saponins being more favorable for foam formation. However, in experimental conditions involving complex saponin extracts, the results in the literature are not always conclusive, and some authors have argued that this classification may not be practical for predicting the foamability and stability of saponins [45].

The foam stability results presented in Table 3 show that QS consistently exhibited the highest foam stability under all conditions. ARS, in some cases, did not show significant differences with QS, indicating its relatively good performance in terms of foam stability. Interestingly, NaCl did not negatively affect QS at the concentration of 0.50 g/100 mL, which might seem surprising given its ionic nature. However, it is important to note that foam characteristics are influenced by various factors, and the specific behavior of saponins can be complex. In general, NaCl tended to decrease foam stability, while sucrose did not have a significant effect. The influence of ionic strength appeared to be more pronounced than that of sucrose on foam characteristics. This aligns with the findings of Chen et al. [47], who tested a saponin extract from *X. sorbifolium* leaves (comprising triterpenic mono- and bidesmosidic saponins) for industrial applications. They observed that the extract exhibited high foam stability in sucrose solutions up to 30%, suggesting its potential use in the formulation of desserts with reduced sugar content, while retaining their rheological and organoleptic characteristics. Foam stability is a complex process influenced by various factors, including saponin chemical structure, concentration, and the presence of other compounds in the extracts. It involves mechanisms such as gravitational drainage, coarsening due to inter-bubble gas transport, and the coalescence of adjacent bubbles [48]. Further studies are needed to fully understand the factors governing foam stability in saponin-containing systems.

### 3.4. Pancreatic Lipase Inhibition Assay

**Figure 8:**
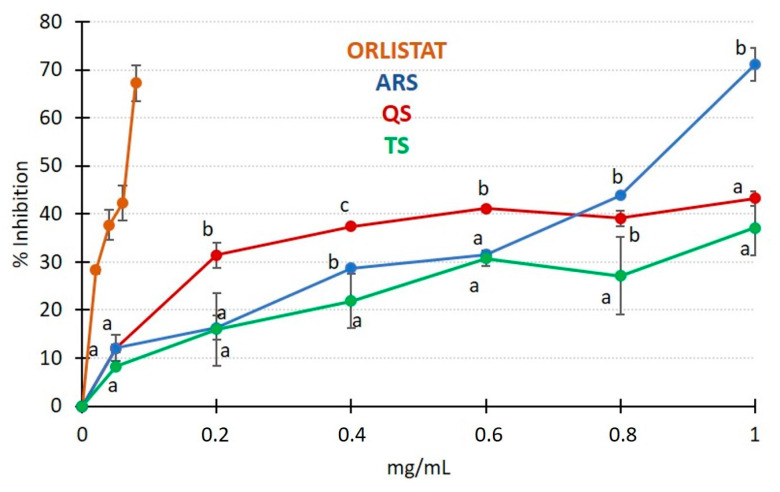
A plot of the percentage of pancreatic lipase inhibition as a function of the concentration (mg/mL) of Orlistat and the various saponin extracts. If the same letter is used to denote different data points, it signifies that there are no significant differences for the same concentration at a significance level of *p* < 0.05.

Pancreatic lipase is a critical enzyme involved in the metabolism of lipids, and substances that inhibit its activity could potentially be useful in addressing obesity and hyperlipidemia [10]. The various extracts studied were tested to assess their inhibitory capacity compared to the only drug approved for obesity treatment, Orlistat. The results of these experiments are presented in Figure 8.

Orlistat clearly demonstrated the highest activity among the substances tested for pancreatic lipase inhibition. In contrast, QS and TS did not exhibit a linear response, and as the concentration increased, a lower inhibitory response was observed. The results of the regression analysis are summarized in Table 4. These statistics allow for the calculation of the IC_50_, which represents the concentration of a substance required to achieve a 50% inhibition of pancreatic lipase. Under the conditions tested, Orlistat had the lowest IC_50_ value (0.058 mg/mL). ARS presented an IC_50_ that was 13 times higher than Orlistat, which is consistent with IC_50_ values reported for saponins from fenugreek (0.59–0.72 mg/mL) [49] and *Acanthopanax sessiliflorus* (0.75 mg/mL) [50]. QS and TS had higher IC_50_ values, at 1.64 and 2.01 mg/mL, respectively, falling within the range reported for other natural extracts from sources such as ginger and buckwheat (1.29–1.94 mg/mL) [9]. These findings provide insights into the inhibitory capacity of the saponin extracts compared to the reference drug, Orlistat, in the context of pancreatic lipase inhibition. The IC_50_ values allow for comparisons between different substances and can guide further research into their potential therapeutic applications in addressing obesity and hyperlipidemia.

The hydrolysis of triglycerides by lipase occurs at the water/oil interface in food emulsions during digestion. Therefore, substances that can decrease the interface area and the surface tension of the oily phase may also act as lipase inhibitors [50]. Saponins have emulsifying capacity, as discussed earlier, and their structural characteristics, along with other accompanying substances, can modulate this activity of natural extracts [33]. In our study, ARS exhibited a lower emulsifying capacity compared to QS. However, its inhibitory activity showed a linear response, resulting in a lower IC_50_ value compared to QS. Several factors could contribute to this observation. The high concentration of saponins in ARS, the diversity of chemical structures (with up to 17 different saponins identified), and the presence of proteins and phenolic compounds in the ARS extract may enhance its lipase inhibitory activity. Some researchers have compared the inhibitory activity of commercial saponins with that of natural extracts [49] and found that commercial saponins often had little to no activity. This discrepancy was attributed to the different structures of saponins present in natural extracts, which may feature different aglycones and/or sugar chains, that could have a synergistic effect. The presence of phenolic compounds in extracts can also contribute to synergistic effects with saponins. In our case, the presence of phenolics, primarily caffeic acid at a concentration of 0.65%, could influence the overall inhibitory activity of ARS. Nevertheless, ARS could be a potential inhibitor of pancreatic lipase, and further in vivo studies are necessary to confirm its activity, as well as to investigate other potential effects such as its hypocholesterolemic activity. Toxicity tests are also essential to assess its safety profile for potential therapeutic applications.

## 4. Conclusions

The saponin-rich extract obtained from asparagus roots through a simple and environmentally friendly patented process can be of great interest to the industry due to its technological characteristics. Asparagus roots are a byproduct of asparagus cultivation that creates significant environmental problems when left in the soil. The application of an easily scalable patent, which does not require high pressures, temperatures, or the use of organic solvents, yields highly rich extracts while maintaining a certain complexity in their chemical composition, which promotes synergies among their different components. Its emulsifying and foaming properties are similar to those of a purified *Quillaja* bark extract, a recognized bio-emulsifier used as a natural additive, making it suitable for formulating “green-label” foods. Furthermore, pH values and the presence of sodium chloride or sucrose in the medium affect its technological properties similarly to how those of *Quillaja* extracts are modified. These features could enable its easy integration into the formulation of carbonated beverages as foaming agents, as emulsifiers in dairy product formulations, and as stabilizers in dairy desserts with a mousse-like texture, with the added advantage of inhibiting fat absorption. In the cosmetics industry, they would also have significant applications as natural surfactants and foaming agents for soaps and shampoos. This extract also exhibits health properties, as it has an inhibitory effect on pancreatic lipase activity, with an IC_50_ lower than that of *Quillaja* and *Tribulus* extracts, suggesting a potential application in anti-obesity and hypocholesterolemic therapies. However, further bioactivity and, especially, toxicity tests are needed to support these uses. The use of these extracts in the food, cosmetic, or pharmaceutical industries would add value to asparagus roots, encouraging the agricultural community to remove them from the soil by simple means, with agricultural machinery similar to potato harvesting, and process them as a source of saponins. This practice would help in addressing the problem of soil autotoxicity in asparagus cultivation and would be a significant step toward the sustainability of this crop, removing this byproduct from the cultivation soil and enhancing its quality and health.

## Figures and Tables

**Table 1:** The chemical composition (g/100 g) of the three extracts assayed (asparagus root saponin extract, ARS; Quillaja saponin extract, QS; Tribulus saponin extract, TS).

	ARS	QS	TS
Saponin	47.64 ^1^	>10 ^2,3^	12.32 ^2^
Phenolics ^1,4^	3.35	1.10	9.42
Protein	10.74 ^1^	0.26 ^1^	4.10 ^2^
Ash	0.60 ^1^	0.90 ^1^	0.22 ^2^

^1^ Determined in our laboratories. ^2^ Stated by product label. ^3^ Content expressed in sapogenin basis. ^4^ Determined by Folin–Ciocalteu method.

**Table 2:** The influence of the presence of sodium chloride and sucrose on the emulsifying capacity and stability of emulsions for ARS, QS, and TS was examined at two concentration levels (0.50 g/100 mL and 0.03 g/100 mL). If the same letter is used to indicate different data points, it means that there are no significant differences for the same concentration (lowercase for comparison of saponin sources and uppercase for comparison of water and additives) at a significance level of *p* < 0.05.

Emulsifying capacity				
Sap. conc.	Saponin	Water	NaCl 1M	Sucrose 5%
0.50	ARS	29.41 ± 0.62 b A	47.69 ± 4.35 b B	30.30 ± 0.64 c A
	QS	32.35 ± 2.08 b B	48.10 ± 3.74 b C	26.13 ± 0.55 b A
	TS	5.88 ± 0.04 a B	29.63 ± 0.74 a C	0.00 ± 0.00 a A
0.03	ARS	18.66 ± 1.06 b B	33.58 ± 1.06 a C	5.88 ± 0.04 b A
	QS	28.36 ± 0.42 c A	44.78 ± 2.11 b B	29.63 ± 0.31 c A
	TS	0.00 ± 0.00 a A	29.85 ± 0.63 a B	0.00 ± 0.00 a A
Emulsion stability				
Sap. conc.	Saponin	Water	NaCl 1M	Sucrose 5%
0.50	ARS	29.85 ± 1.06 b B	7.46 ± 0.21 b A	28.79 ± 2.14 b B
	QS	31.11 ± 0.33 b A	40.60 ± 0.43 c B	31.82 ± 0.43 b A
	TS	0.00 ± 0.00 a A	0.00 ± 0.00 a A	7.65 ± 0.11 a B
0.03	ARS	22.22 ± 0.23 b C	0.00 ± 0.00 a A	7.63 ± 0.08 b B
	QS	28.15 ± 0.29 c B	0.00 ± 0.00 a A	27.27 ± 0.43 c B
	TS	0.00 ± 0.00 a A	0.00 ± 0.00 a A	0.00 ± 0.00 a A

**Table 3:** The influence of the presence of sodium chloride and sucrose on the foamability and foam stability of ARS, QS, and TS at two concentration levels (0.50 g/100 mL and 0.03 g/100 mL). If the same letter is used to indicate different data points, it indicates that there are no significant differences for the same concentration (lowercase for comparison of saponin sources and uppercase for comparison of water and additives) at a significance level of *p* > 0.05.

Foaming capacity				
Sap. conc.	Saponin	Water	Sodium chloride 1M	Sucrose 5%
0.50	ARS	46.67 ± 4.71 b A	46.67 ± 1.89 b A	45.00 ± 2.36 c A
	QS	43.33 ± 4.71 b B	26.67 ± 3.30 a A	27.47 ± 2.30 b A
	TS	28.33 ± 2.36 a B	25.00 ± 2.83 a B	13.33 ± 0.94 a A
0.03	ARS	20.00 ± 2.36 b A	33.33 ± 2.83 b B	25.00 ± 1.89 b A
	QS	28.36 ± 0.42 c B	38.33 ± 2.36 b C	23.33 ± 1.41 b A
	TS	0.00 ± 0.00 a A	13.33 ± 0.94 a C	6.67 ± 0.47 a B
Foam stability				
Sap. conc.	Saponin	Water	Sodium chloride 1M	Sucrose 5%
0.50	ARS	53.33 ± 4.71 b B	35.74 ± 1.44 b A	51.92 ± 2.72 b B
	QS	61.91 ± 6.74 c A	75.58 ± 9.35 c A	68.73 ± 0.33 c A
	TS	47.22 ± 3.93 a B	33.12 ± 3.80 b A	37.34 ± 4.43 a AB
0.03	ARS	74.83 ± 2.97 b B	24.32 ± 4.30 b A	73.19 ± 3.91 b B
	QS	71.84 ± 2.61 b B	64.96 ± 8.30 c A	71.56 ± 4.34 b B
	TS	0.00 ± 0.00 a	0.00 ± 0.00 a	0.00 ± 0.00 a

**Table 4:** Regression analysis of results obtained from pancreatic lipase inhibition assays and the IC_50_ concentration (mg/mL) calculated from regression models.

	Regression Model	R^2^ (%)	a	b	IC_50_ (mg/mL)
Orlistat	$y={\left(a+b\sqrt{x}\right)}^{2}$	95.70	0.4517	27.5093	0.058
ARS	y=a+bx	93.22	3.2900	59.2210	0.7887
QS	$y=\surd (a+b\sqrt{x})$	93.77	−37.0366	1983.12	1.6366
TS	y=a+b√x	96.54	0.2352	35.0949	2.0107

Orlistat: positive control; a: intercept; b: slope; IC_50_: concentration at 50% inhibition.

## Data Availability

Data is contained within the article.
